# Production of serine protease inhibitors by mutagenesis and their effects on the mortality of *Aedes aegypti* L. larvae

**DOI:** 10.1186/s13071-015-1127-4

**Published:** 2015-10-06

**Authors:** Tatiane Sanches Soares, Ricardo José Soares Torquato, Yamile Gonzalez Gonzalez, Francisco Jose Alves Lemos, Aparecida Sadae Tanaka

**Affiliations:** Departamento de Bioquímica, Escola Paulista de Medicina, Universidade Federal de São Paulo, Rua 3 de Maio 100, 04044-020 São Paulo, SP Brazil; Laboratório de Biotecnologia, Universidade Estadual do Norte Fluminense, Rio de Janeiro, RJ Brazil

**Keywords:** Mosquito, Serine protease inhibitor, Larvae, *Aedes aegypti*, Larvicide

## Abstract

**Background:**

Dengue, transmitted primarily by the bites of infected *Aedes aegypti* L., is transmitted to millions of individuals each year in tropical and subtropical areas. Dengue control strategies are primarily based on controlling the vector, using insecticides, but the appearance of resistance poses new challenges. Recently, highly selective protease inhibitors by phage display were obtained for digestive enzymes of the 4th instar larvae (L4) midgut. These mutants were not confirmed as a larvicide due to the low yield of the expression of these inhibitors. In the present study, chimera molecules were constructed based on the mutations at positions P1-P4’ selected previously. The T6, T23 and T149 mutants were mixed with another Kunitz inhibitor, domain 1 of the inhibitor boophilin (D1).

**Methods:**

The chimeras T6/D1, T149/D1 and T23/D1 were expressed at high levels in *P. pastoris* yeast, purified by ionic exchange chromatography and their homogeneity was analyzed by SDS-PAGE. The chimera inhibitors were assayed against larval trypsin, chymotrypsin and elastase using specific chromogenic substrates. The inhibitors were assayed for their larvicide potential against L4.

**Results:**

The chimeras exhibited strong inhibitory activities against the larval digestive enzymes in a dose-dependent manner. T6/D1, T149/D1 and T23/D1 exhibited strong larvicidal activity against L4 of *Ae. aegypti* with inhibitor concentrations in the μM range. A synergistic increase in mortality was observed when a mixture of the three chimeric inhibitors was tested.

**Conclusions:**

The strategy for constructing the chimeric inhibitors was successful. The chimeras showed strong larvicidal activity against *Ae. aegypti*. In the future, our findings can be used to design synthetic inhibitors for larvae digestive enzymes as an alternative method to control the dengue vector.

## Background

*Aedes aegypti* (Linnaeus) is the primary vector of several arboviruses including dengue, yellow fever and chikungunya [1, 2]. *Aedes aegypti* is well adapted to urban areas, and represents a major problem for public health [2]. An estimated 500,000 people with severe dengue are hospitalized each year, and approximately 2.5 % die [3]. Unfortunately, there is no vaccine or treatment for dengue available, and vector reduction remains the main method for reducing the transmission of the dengue virus [3].

Digestive enzymes present in *Ae. aegypti* larvae include trypsin-like, chymotrypsin-like and elastase-like serine proteases [4]. A transcriptional analysis of the trypsin-like enzymes present in the midgut of laboratory-reared *Ae. aegypti* was performed, and the key enzyme, a native trypsin, was purified and characterized [4].

The trypsin inhibitor from *Moringa oleifera* flowers (MoFTI) interferes with the survival and development of *Ae. aegypti* larvae. Mortality of newly hatched larvae (L1) was observed in the presence of the *M. oleifera* flower extract and MoFTI [5]. A lectin from *M. oleifera* seeds (WSMoL) was shown to kill *Ae. aegypti* L4 by causing morphological alterations in the digestive tract and inhibition the digestive enzymes [6].

Recently, phage display was performed using a selection of inhibitors to the larvae digestive enzymes [7]. A library of mutants was constructed using the inhibitor HiTI as a template, and the reactive site region, P1 to P4', of the inhibitor was randomized. The HiTI mutants that showed strong inhibitory activity were T6 for trypsin and T23 and T149 for both chymotrypsin and elastase. As the HiTI mutants’ expression in yeast was very low, it was not possible to evaluate their larvicidal activity.

The strategy adopted in this work was to insert the mutations at positions P1 to P4' of HiTI, T6, T23, and T149, into domain 1 of the thrombin inhibitor boophilin [8], which exhibited high levels of expression in *P. pastoris* yeast. The new chimera molecules were designated mutants T6/D1, T23/D1, and T149/D1 and were produced and tested as larvicide for *Ae. aegypti* larvae. As the mosquito larvae is confined to and continuously feeds in aquatic environments [9], inhibition of the digestive enzymes present at this stage of life could be a useful strategy to control the mosquito populations.

## Methods

### Cloning and expression of the inhibitors in *Pichia pastoris*

**Fig. 1:**
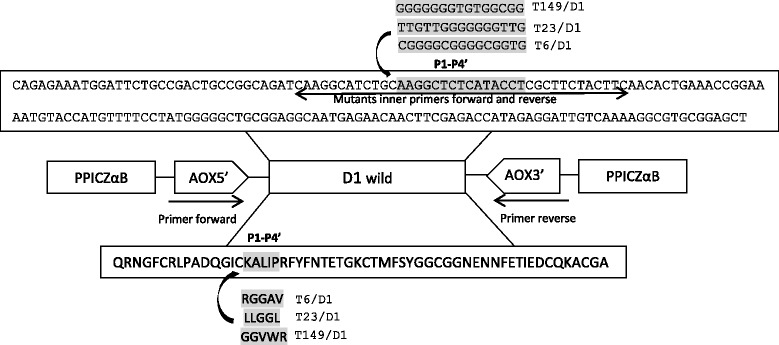
The strategy for the construction of the chimeric inhibitors sequences using the construction of the inhibitor boophilin domain 1 (D1) in pPICZαB plasmid as a template. Nucleotides sequence of wild D1, the region used to construct internal oligonucleotides is represented by left right arrow. Sequence nucleotides and amino acids in region P1-P4’ of the D1 inhibitor that was replaced in production of mutants are shaded in gray. AOX5’ is the primer forward present in plasmid pPICZαB and AOX3’ the reverse primer. The internal primer to construct the mutants were designer in the same place, the internal reverse primer is matched with AO5’ in PCR reaction to form the first part of the inhibitor and internal forward primer is matched with AOX3’ to form the second part of the inhibitor, this parts were joined by a PCR reaction using the AOX5’and AOX3’ primers

For this work, we constructed chimeric inhibitors in which the region P1-P4’ of the selected inhibitors by phage display previously [7] was cloned substituting this region in the domain 1 of the boophilin inhibitor (D1) (Fig. 1), which has a high level of protein expression. As the boophilin inhibitor domain 1 (D1 wild) had been cloned previously in our laboratory in pPICZαB plasmid this construction D1 wild/pPICZαB was used as a template for PCR reactions. To replace the P1-P4’ region (reactive site) of the inhibitor boophilin D1 by this region of the inhibitors T6, T23 and T149, were designed internal oligonucleotides flanking this region for each inhibitor. The internal oligonucleotides sequences used were: T6D1IntFw 5’-CAAGGCATCTGCCGCGGTGGTGCCGTGCGCTTCTACTTC-3’; T6D1IntRv 5’-GAAGTAGAAGCGCACGGCACCACCGCGGCAGATGCCTTG-3’; T23D1IntFw 5’-CAAGGCATCTGCCTACTAGGTGGTCTACGCTTCTACTTC-3’; T23D1IntRv 5’-GAAGTAGAAGCGTAGACCACCTAGTAGGCAGATGCCTTG-3’; T149D1IntFw 5’-CAAGGCATCTGCGGTGGTGTGTGGCGCCGCTTCTACTTC-3’ e T149D1IntRv 5’-GAAGTAGAAGCGGCGCCACACACCACCGCAGATGCCTTG-3’. The DNA amplifications by PCR were performed in two steps: firstly, DNA was amplified the initial region of the inhibitors until the reactive site containing the substitutions (AOX5’ and D1IntRv oligonucleotides), and secondly, it was amplified the region of the reactive site containing the substitutions until the end of the inhibitors (D1IntFw and AOX3’ oligonucleotides), construction D1 wild/pPICZαB was the template for both. Afterwards, the two DNA fragments were united by a PCR reaction using primers AOX5’ and AOX3' flanking region of the initial and end of the inhibitors, this procedure was made to each mutant. Purified DNA fragments were digested with *Xho*I and *Not*I restriction enzymes and ligated into the pPICZαB vector, (Invitrogen, Carlsbad, CA, EUA) previously digested with the same restriction enzymes. The resulting constructs were used in the transformation of bacteria *E. coli* DH5-α, purified, sequenced and verified. The constructs were used to transform yeast *P. pastoris* strain GS115 by electroporation. To identify positive yeast clones expressing each of the inhibitors, five clones from *P. pastoris* strain GS115 containing the insert of each inhibitor, identified and verified by PCR (AOX5’ and AOX3’ oligonucleotides), were individually inoculated in 2.5 mL of BMGY medium (Buffered Glycerol-complex Medium) (1.0 % (w/v) yeast extract, 2.0 % (w/v) peptone in 100 mM potassium phosphate buffer, pH 6.0, 1.34 % (w/v) YNB, 4 × 10^−5^% (w/v) biotin and 1 % (v/v) glycerol) in a 50 mL sterile tube and incubated at 30 °C for 28 h at 250 rpm. The yeast cells were harvested by centrifugation at 3000 × *g* for 5 min at 4 °C, and resuspended in BMMY medium (Buffered Methanol-complex Medium) (BMGY with glycerol replaced by 0.5 % (v/v) methanol) to an absorbance of 1.0 at 600 nm. Proteins were expressed at 30 °C with shaking at 250 rpm, for 96 h with the addition of 0.5 % (v/v) methanol every 24 h. After removing the cells by centrifugation (4000 × *g* for 20 min at 4 °C), the supernatants were assessed for their ability to inhibit bovine chymotrypsin, bovine trypsin or neutrophil elastase using enzymatic assays and the following specific chromogenic substrates: Tosyl–Gly–Pro–Arg–pNA for trypsin, Suc–Ala–Ala–Pro–Phe–pNA for chymotrypsin and Suc–Ala–Ala–Pro–Val–pNA for elastase. Individual clone expressing high levels of each inhibitor was selected. A single *P. pastoris* colony (Mut+) expressing high level of the mutants T6/D1, T23/D1 T149 and D1 wild was selected and used to inoculate 120 mL of BMGY medium in a 1 L sterile flask, which was then further incubated at 30 °C and 250 rpm for 24 h. Protein expression was performed as described above, and the supernatant of the culture was stored at −20 °C.

### Purification of the inhibitors T6/D1, T23/D1, T149/D1 and D1 wild

The expression supernatants (~1 L) of the inhibitors T6/D1, T23/D1, T149/D1 and D1 wild were dialyzed with 0.04 M Tris–HCl, pH 8.0, and applied to ion exchange chromatography on HiTrap Q column. The inhibitors (~200 mL supernatant per chromatography) were purified using ion exchange chromatography on HiTrap Q column (GE, Fairfield, CT, EUA), connected to a medium pressure chromatography system ÄKTA Prime, previously equilibrated with 0.05 M Tris–HCl, pH 8.0. After washing, the proteins were eluted using a NaCl linear gradient (0–1 M) in 0.05 M Tris–HCl, pH 8.0, at flow rate 1.5 mL / min for 60 min. Flow through and eluted fractions from the HiTrap Q column were tested for inhibitory activities to trypsin (T6/D1 and wild D1) and elastase (T23/D1 and T149/D1) using the chromogenic substrates Tosyl-Gly-Pro-Arg-pNA, and Suc-Ala-Ala-Pro-Val-pNA, respectively. Purified inhibitors were concentrated and analyzed by 15 % SDS-PAGE gels [10].

### Larvicidal activity assay using the mutants T6/D1, T23/D1, T149/D1 and D1 wild

Adult *Ae. aegypti* colonies (Rockefeller strain) were reared in cages, at 27 °C and provided of 10 % sucrose solution in distilled water. A glass container filled with distilled water was kept inside each cage to maintain the humidity. Larvae were maintained in distilled water and fed on a minced commercial mouse food. The fourth (L4) instar larvae were used in the experiments. For the assay, groups of 15 L4 were placed in 5 mL of distilled water containing the following total protein concentrations of the mutants T6/D1 (111–588 μM), T23/D1 (79–858 μM), T149/D1 (64–684 μM) and D1 wild (64–684 μM). The lethal concentrations required for killing 50 % of *Ae. aegypti* larvae (LC_50_) after 24 and 48 h were calculated by probit analysis with a reliability interval of 95 % using the StatPlus 2006 software (AnalystSoft, Vancouver, BC, Canada). The experiments were performed in triplicate and monitored at 24 h and 48 h. ANOVA (*p* < 0.05) and the Tukey's test used in the statistical analysis were calculated using GraphPad Prism version 4.0 for Windows (GraphPad Software, San Diego, CA, USA).

### Inhibitory activity assay of mutant inhibitors T6/D1, T23/D1, T149/D1 for larvae serine proteases

Larvae midgut extract (0.8 mg total protein was used for trypsin and chymotrypsin and 24.5 mg for elastase) was pre incubated with different concentrations of the inhibitors T6/D1, T23/D1, T149/D1 in 0.1 M Tris/HCl (pH 8.0) containing 0.1 % Triton X-100 for 10 min at 37 °C. Afterwards, residual enzymatic activities were measured at 405 nm by adding Suc-Ala-Ala-Pro-Val-pNA, Tosyl-Gly-Pro-Arg-pNA and Suc-Ala-Ala-Pro-Phe-pNA as chromogenic substrates for neutrophil elastase, trypsin and chymotrypsin, respectively.

## Results

### Cloning, expression and purification of the chimeric inhibitors

**Fig. 2:**
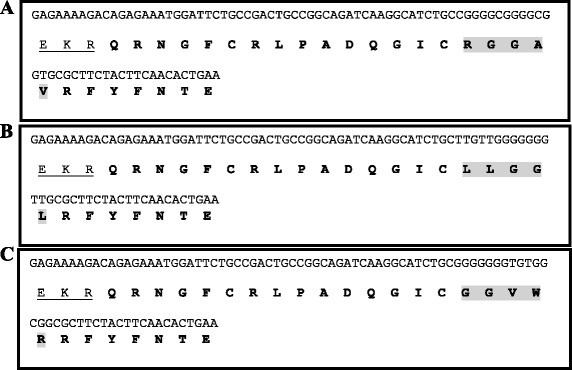
Nucleotides and traduced amino acid sequences of the chimera cloned in the pPICZαB vector. **a**. Mutant T6/D1; **b**. Mutant T23/D1; **c**. Mutant T149/D1. The sequence of pPICZαB vector is underlined, and the mutated regions (P1-P4’) of the inhibitors are shown in gray

**Fig. 3:**
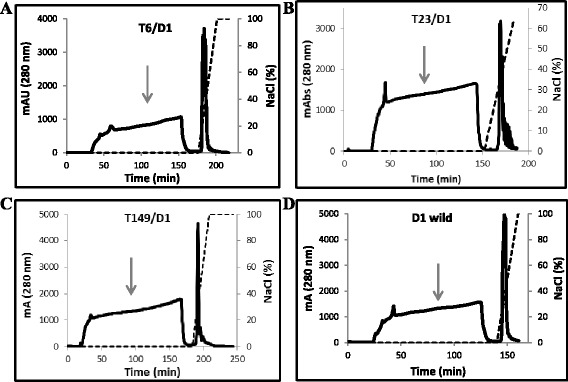
Purification of the chimeras T6/D1, T149/D1, T23/D1 and D1 wild expressed in P. pastoris by ion exchange chromatography in HiTrap Q column. The supernatant of the culture medium dialyzed with 0.05 M Tris/HCl pH 8.0 buffer was applied in the column pre equilibrated with the same buffer. The elution of proteins was performed with a linear gradient of NaCl (0–1 M). The regions containing inhibitory activities are indicated by gray arrows

**Fig. 4:**
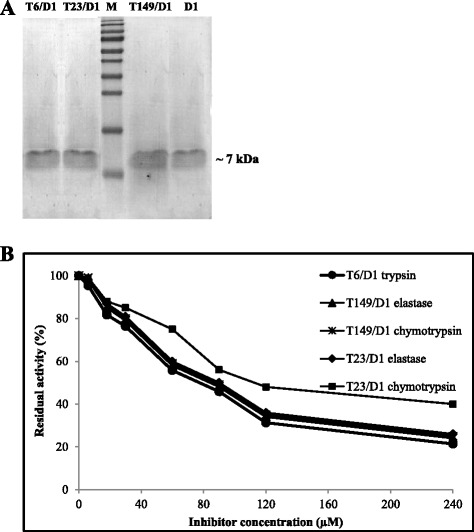
Characterization of the mutants: T6/D1, T23/D1, T149/D1 and D1 wild. **a** SDS–PAGE (15 %) of the flow through of the ion exchange chromatography in HiTrap Q column of the mutants T6/D1, T23/D1, T149/D1 and D1 wild. **b** Inhibition assay of the chimeric inhibitors for larvae serine proteases. Enzymatic activities using the L4 midgut extract of *Ae. aegypti* and chromogenic substrate for trypsin (Tosyl-Gly-Pro-Arg-pNA), chymotrypsin (Suc-Ala-Ala-Pro-Phe-pNA) and elastase (Suc-Ala-Ala-Pro-Val-pNA). Both trypsin and chymotrypsin assays were performed using 0.8 mg total protein, and 24.5 mg total protein for elastase

Amino acids at positions P1-P4' of D1 (KALIP) were replaced by the amino acids presented at those positions in the mutants T6 (RGGAV), T23 (LLGGL) and T149 (GGVWR), selected previously by phage display. The nucleotide sequences of T6/D1 (Fig. 2a), T23/D1 (Fig. 2b), and T149/D1 (Fig. 2c) clones in the pPIZαB vector were confirmed by PCR amplification followed by DNA sequencing. Mutants T6/D1 (Fig. 3a), T23/D1 (Fig. 3b), T149/D1 (Fig. 3c) and wild D1 (Fig. 3d) were purified by ion exchange chromatography and by SDS-PAGE confirmed the homogeneity of the inhibitors (Fig. 4a).

### Inhibitory activity of the mutants for larval serine proteases

All mutants showed inhibitory activity against larvae digestive enzymes in a dose-dependent manner (Fig. 4b). T6/D1 was able to inhibit larval trypsin-like enzymes, and T23/D1 and T149/D1 both inhibit chymotrypsin-like and elastase-like enzymes.

### Larvicidal assays using mutants T6/D1, T23/D1, T149/D1 and wild D1

**Fig. 5:**
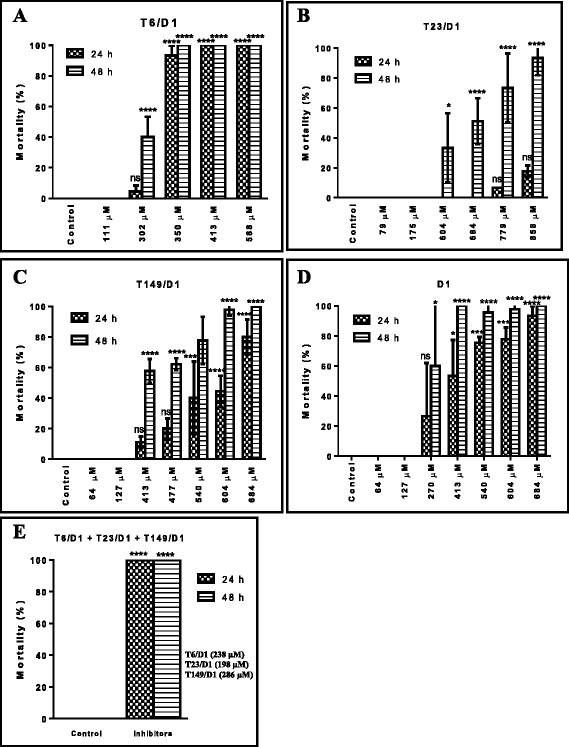
Larvicidal effect of chimeric inhibitors against L4 *Ae. aegypti* larvae*,* in 24 h and 48 h assays. **a**. Mutant T6/D1; **b**. Mutant T23/D1; **c**. Mutant T149/D1; **d**. D1 wild; **e**. Assay with combination of the mutants T6/D1, T23/D1 and T149/D1. The experiments were performed in triplicate using experimental group containing 15 larvae each. The larval mortality level of the inhibitors was significant increase when compared to control (*p < 0.05, **p < 0.01, ***p < 0.001, ****p < 0.0001 and ns-not significant, in Tukey’s Multiple Comparison Test). ANOVA (*p* ≤ 0.05) and Tukey's analysis were used for statistical analysis. Error bars correspond to the standard error of the mean of three experiments

Larvicidal potential of the mutant inhibitors and wild D1 were evaluated on L4 larvae. The mutant T6/D1 showed 100 % of mortality against L4 at 350 μM after 24 h. At 302 μM, mutant T6/D1 showed approximately 40 % mortality in L4 larvae after 48 h (Fig. 5a). The LC_50_ values determined for T6/D1 were of 380 μM and 302 μM for larvae after 24 h and 48 h of assay, respectively. The mutant T23/D1 presented mortality of 35-95 % in concentrations between 604 μM to 858 μM, at 48 h (Fig. 5b). The LC_50_ values for T23/D1 were of 1.1 mM and 611 μM for larvae after 24 h and 48 h, respectively. At 24 h, the mutant T149/D1 showed 18-80 % mortality of L4 larval between the concentrations 413 μM to 684 μM. At 48 h, mortality increased to 60-100 % (Fig. 5c). The LC_50_ values for T149/D1 were of 577 μM and 421 μM for larvae after 24 h and 48 h, respectively. A synergic effect was observed when mutant T6/D1 (238 μM), T23/D1 (198 μM) and T149/D1 (286 μM) were mixed together and tested against L4 *Ae. aegypti* larvae, at concentrations that individually none of the inhibitors presented larvicidal effect, all together showed 100 % mortality (Fig. 5e).

The wild D1 showed larval mortality of 27-93 % at 270 μM to 684 μM, respectively at 24 h. The estimated LC_50_ for D1 were of 474 μM for larvae after 24 h. At 48 h, the L4 mortality was 60-100 % (Fig. 5d). All inhibitors were used in assays against L1, at the same concentrations used in the assays for L4 *Ae. aegypti* larvae, but larvicidal activity was not observed (data not showed).

## Discussion

The advance in integrated pest management has been caused by most cost-effective methods and common-sense practices for control of pests that represent a low hazard to the environment and people [11]. Phage display is a powerful technique used to study protease inhibitors [12–14]. Recently, our group used phage display system and the inhibitor HiTI [15] to select specific inhibitor for digestive enzymes of *Ae. aegypti* larvae [7]. In this work, based on data of Soares et al. [7], the mutated regions (residues P1-P4’) of selective HiTI mutated inhibitors identified for digestive enzymes *Ae. aegypti* larvae were inserted into another Kunitz domain inhibitor, to increase the level of recombinant protein expression. The template inhibitor selected was the boophilin inhibitor domain 1 (D1) of *Rhipicephalus microplus.* Previously, boophilin domain 1 had been cloned and expressed by our group and shown to a high protein expression level of approximately 20 mg/L [8]. The chimeric molecules, named mutants HiTI T6, T23 and T149, were selected by strong inhibition activity of digestive enzymes of *Ae. aegypti* larvae, presenting IC_50_ in nM range [7].

The continuous and indiscriminate use of synthetic insecticides has led to emergence resistant *Ae. aegypti* populations, environmental persistence and unselective toxicity [16]. Natural insecticides have been investigated for larvicidal effect on *Ae. aegypti* resistant strains because they are usually available at low cost, are usually highly biodegradable, and the larvae usually do not develop cross-resistance to them, minimizing resistance development [17]. Thus, alternative methods and new strategies for vector control need to be developed. Purified proteins from plants have been demonstrated larvicidal activity against *Ae. aegypti*. Many plant insecticides primarily target the midgut of the mosquito larvae, and some are able to interfere with the larval development into adult stage. A trypsin inhibitor (MoFTI) purified from *Moringa oleifer flowers* caused mortality of hatched newly larvae [5]. And, a lectin extracted from *M. oleifera* seeds (WSMoL) was able to kill L4 *Ae. aegypti* by causing morphological alterations in the larvae digestive tract and inhibition digestive enzymes [6]. A lectin from *Myracrodruon urundeuva* leaf showed effect on survival and digestive enzymes activity from *Ae. aegypti* larvae [18]. Essential oils from Brazilian Croton species showed larvicidal activity against *Ae. aegypti* [16]. So, the production of highly selective inhibitors for the digestive enzymes from *Ae. aegypti* larvae by mutagenesis presents itself as an innovative approach in this area of study.

The enzymes present in the gut of larvae of *Ae. aegypti* responsible for its digestion are trypsin-like, chymotrypsin-like and elastase-like serine proteases [7, 19]. In the present work, the mutant T6/D1, a trypsin inhibitor, in the concentration of 350 μM caused the highest mortality (100 %) in L4 *Ae. aegypti*. This results corroborate the data that, trypsin is the major enzyme presents in the midgut of *Ae. aegypti,* being the main responsible for digestion in larvae [7] and the impairment of its activity may result in poor nutrient absorption and non-availability of essential amino acids. The mutant T23/D1, also showed larvicidal effect against L4, but its effect was only observed after 48 h with mortality rates of 50-93 %. The mutant T149/D1 showed larval mortality of 60-100 % at 48 h of exposure. T149/D1 was more effective as larvicide than T23/D1. These data support our hypothesis that T149/D1 may have been selected not only to the digestive enzymes but also to other molecules present in the larval midgut that are important for the larvae larval physiology. As T23/D1 and T149/D1 are inhibitors of the chymotrypsin and elastase enzymes that are apparently in low concentration in the larvae, which are in consonance with lower larvicidal effect than T6/D1.

In an attempt to evaluate the synergic effect in the inhibition of all enzymes present in midgut, the three mutants T6/D1, T23/D1 and T149/D1 were added in a larvicidal assay. Surprisingly, the concentration of each inhibitor used in the blend not caused mortality when used separately, but in combination, they caused 100 % L4 mortality. Confirming that, the inhibitors efficiency was higher when all digestive enzymes present in the midgut were inhibited in the same time. None of the three mutants presented larvicidal effect against L1 *Ae. aegypti*, confirming the specific-targeting power of phage display selection system to effect only L4 larval.

To remove any doubt regarding the specificity of the inhibitors in the larvicidal assay, the wild D1 inhibitor was used in the larvicidal experiments. However, only wild D1 caused mortality of approximately 50 % of L4 compared with 100 % mortality obtained for the T6/D1 and not mortality for the T23/D1, at the same concentration. D1 was used as a control since only its reactive site was removed for construction of chimeras. As inhibitor D1 wild is a strong bovine trypsin inhibitor, it was expected some effect on larval survival, but the results support our conclusion that the mutant selection was specific for the 4th larval instar and that trypsin-like enzymes are important for larval physiology.

For the first time highly selective inhibitors have been selected specifically for digestive enzymes from *Ae. aegypti* larvae. The chimeric inhibitors may have impaired digestive and absorption processes in the midgut of *Ae. aegypti* larvae compromising their survival.

## Conclusions

Our findings indicate that the mutants selection by phage display were specific for the L4 *Ae. aegypti*. The mutant inhibitors were efficient in causing larval mortality; and the synergism of mutant inhibitors selected for different digestive enzymes was more effective as larvicide. In the future, these inhibitors can be used in control measures for the larvae of *Ae. aegypti* mosquito.

## Abbreviations

D1: Boophilin inhibitor domain 1
T6/D1, T23/D1, and T149/D1: Mutants of the boophilin inhibitor domain 1
